# Does the Graphic Health Warning on Tobacco Products Have an Influence on Tobacco Consumers in India? A Scoping Review

**DOI:** 10.7759/cureus.38304

**Published:** 2023-04-29

**Authors:** Abhay Mudey, Apoorva Shukla, Sonali G Choudhari, Abhishek Joshi

**Affiliations:** 1 Community Medicine, Jawaharlal Nehru Medical College, Datta Meghe Institute of Higher Education and Research, Wardha, IND; 2 Preventive Medicine, School of Epidemiology and Public Health, Datta Meghe Institute of Higher Education and Research, Wardha, IND; 3 Community Medicine, School of Epidemiology and Public Health, Jawaharlal Nehru Medical College, Datta Meghe Institute of Higher Education and Research, Wardha, IND

**Keywords:** cigarettes and other tobacco products act (cotpa), india, smokeless tobacco, graphic health warning, tobacco

## Abstract

It is now a legal requirement in India that all tobacco products carry graphic health warnings on their packaging. The Government of India has taken steps to reduce the number of people using tobacco products by introducing the Cigarettes and Other Tobacco Products Act. This law states that all tobacco products must display health warnings on their packaging. Despite this, many people in India still use tobacco products. Graphic health warnings on tobacco packaging and labeling can be an effective way to spread awareness about the risks associated with smoking. Pictures or pictograms should be included on all products, as this can be particularly helpful for those with limited literacy skills. This way, everyone can understand the dangers of tobacco use, even if they cannot read.

This review examines the effectiveness of pictures on cigarette and other tobacco product packages as health warnings in India. To assess the impact of these graphic warnings, research from India was reviewed, along with information from the Ministry of Health and Family Welfare websites and the World Health Organization. The results indicate that the images on packages have some positive effects. However, they need to be made more visible and easier to comprehend to have a more significant influence on discouraging people from taking up smoking.

## Introduction and background

Since its discovery, tobacco has been humanity's leading and most dangerous killer. Most tobacco stakeholders, including caregivers, producers, and tobacco farmers, as well as the majority of consumers, suffer from multiple chronic health hazards. This chronic health hazard shortens their life span and leads to miserable death [1]. Cigarette smoke consists of approximately 4,000 different chemicals, many of which are active and can cause harm to the body, including toxicity, mutation, and cancer. Nicotine, the main ingredient in tobacco, is so addictive that it can cause an addiction comparable to that of heroin [2].

The high availability of tobacco products in India contributes to the country's high tobacco consumption rates. Cigarettes, beedis, and hookah are all products that can be smoked while chewing; khaini, gutkha, or pan masala are all examples of smokeless tobacco products [1-3]. Tobacco use leads to a variety of physical effects, such as stains on the teeth, gum disease, and tooth loss. Additionally, it can result in a serious form of cancer known as oral cancer. This is often preceded by red and white lesions, which can be an early sign of cancer [4,5].

The World Health Organization (WHO) has declared that there are six MPOWER strategies to combat tobacco use, and one of those strategies is to have graphic health warnings [1,6]. The use of pictures to warn people about the health risks of tobacco use is an effective way to make people aware of the dangers of tobacco use and encourage them to change their behavior, such as by quitting or reducing tobacco consumption. The large, colorful, and frightening images on tobacco products serve as a warning to both users and non-users of the dangers of tobacco use [7]. Tobacco packaging featuring graphic health warnings is an effective way to spread information about the risks of smoking. These warnings are easy to notice due to their prominent placement on the packages. Furthermore, they cost very little to implement and reach a wider audience than other methods of informing people about the risks of smoking. By keeping these messages visible on the packaging, tobacco users are more likely to pay attention to the health warnings and understand the risks associated with smoking [8].

This review seeks to evaluate the impact of graphics and text printed on tobacco product packaging in India. The review will consider how well people understand graphic health warnings, their effects on tobacco users, the messages contained in the warnings, how they affect people's decisions to quit smoking, and any potential negative outcomes.

This review is important for primary care practice because it can help with early screening and prompt diagnosis of tobacco-related health problems. It also covers topics relevant to patients or people with a history of tobacco use, doctors, physicians, public health professionals, and teenagers who might be under peer pressure to use tobacco.

## Review

Methodology

The PubMed and Google Scholar databases were searched for relevant results. To identify relevant “grey literature,” electronic searches were also conducted. The Ministry of Health and Family Welfare (MoHFW) website and WHO database were reviewed to locate studies conducted in India that were relevant to our research. We used the reference lists of relevant articles to find more studies, and all research was verified by using additional criteria, such as if the article was available in full text for free. We used keywords such as "health warning," "health message," "health communication," "label," and "labelling," in combination with "smoking," "tobacco," "cigarette," and "smokeless tobacco," to identify relevant articles.

Global and Indian figures

Tobacco consumption is a global issue with significant health implications. Every year, 8 million people around the world die from issues related to tobacco use. The majority of these deaths (7 million) result from using tobacco, while the other 1.2 million come from being exposed to second-hand smoke. Most of those who use tobacco, around 1.3 billion people, are from low and middle-income countries. In 2020, a total of 22.3% of the world's population were tobacco users, with 36.7% of men and 7.8% of women [9-11]. All in all, tobacco use is one of the major preventable causes of death worldwide [5].

In India, nearly 267 million adults consumed tobacco in 2016-17, making up almost a third of the population. This habit caused an economic burden of INR 177,341 crore (or USD 27.5 billion) due to tobacco-related illnesses among those aged 35 and up in 2017-2018, as revealed in the Global Adult Tobacco Survey (GATS), India [11-13]. Smoking has long been linked to a number of diseases, and new evidence suggests a link between smoking and five additional diseases in adults: age-related macular degeneration, diabetes mellitus, tuberculosis, liver cancer, and colorectal cancer. While smoking can weaken the immune system and reduce cancer survival rates, the exact magnitude of this effect is unknown, and the number of smoking-related deaths from these conditions is relatively low when compared to lung cancer, particularly in younger people.

WHO's Framework Convention on Tobacco Control recommends the use of pictures on cigarette packaging in order to display health warnings. According to the treaty, such graphic warnings are more effective at decreasing smoking rates than warnings that are only composed of text. As a result, 7 nations and regions have adopted this policy, and it impacts approximately half of the world's population [9,14].

Laws and legislation for tobacco control

Article 11 of the WHO Framework Convention on Tobacco Control (FCTC 2003) requires that any health warnings on cigarette packs be clear and highly visible. They must occupy at least 30% of the front and back of the package and should take up at least half of the primary surface area. It is further suggested that the warnings be changed regularly and should feature images as well as text [14,15]. The Government of India has made a concerted effort to reduce the number of people using tobacco products. In 2003, the Cigarettes and Other Tobacco Products Act (COTPA) was passed, which requires that 40% of the front of all tobacco product packages be covered with pictures of health warnings. This is intended to alert people to the potential dangers of using such products. Additionally, public smoking has been banned, and the advertising of tobacco products is prohibited [14,16,17].

In 2006, the Indian government first mandated that all tobacco products feature graphic warnings. These warnings became enforceable on May 31, 2009, under COTPA [15]. On May 27, 2009, the government released a series of graphic health warnings that required different levels of implementation. After numerous modifications, these warnings were required to cover 85% of the front and back of tobacco product packages, with separate warnings for products people smoke and those they do not, as of April 1, 2015 [14,18]. As of 2018, more than 100 countries have implemented graphic warnings on cigarette packages [14,19].

COTPA states that when selling tobacco products, warnings must be presented in both the language in which the brand name appears as well as any other language commonly used in the area of sale. This ensures that everyone has access to health warnings regardless of language. Most tobacco brands do not have warnings in local or regional languages, and they limit supportive text warnings to English literates due to the vague specification of COTPA [6,20].

Smokeless tobacco

It was found that smokeless tobacco products were used by 21.4% of the population (199.4 million), and an interesting observation was that more women used these products than combustible ones [21,22]. At this time, there is not much research supporting the use of health warnings for any other forms of tobacco besides cigarettes, such as smokeless tobacco, "fine cut" or "loose" tobacco, water pipes, and beedis [23]. The lack of standardization in smokeless tobacco products' packaging makes it difficult to distinguish the primary surface area. Additionally, due to the packaging's range of shapes and forms, health warnings may need to be tailored to each product. This presents a challenge in terms of ensuring that consumers are aware of the potential health risks associated with the product [22,23]. A 2016 study conducted by Gravely et al. on four states of India showed that health warnings on smokeless tobacco packages were not very effective, and even changing from the symbolic warning (prior to the policy) to graphic health warnings (after the policy) did not lead to any evident increases in effectiveness among those who still used smokeless tobacco products. This implies that just changing the image is not enough to have an effect [24,25].

Perceived influence of graphic warning

Tobacco is a unique consumer product in that it can cause harm to everyone who is exposed to it, not just those who use it. It is estimated that up to half of those who use tobacco as intended will eventually die from it. Despite this, tobacco use remains prevalent due to its low cost, aggressive and widespread marketing, lack of knowledge about the risks associated with it, and weak or non-existent regulations prohibiting its use [26,27].

One of the most economical ways to make people aware of the dangers of smoking is to add graphic images of the health risks to the packaging of all tobacco products. This will make both current smokers and those who do not smoke aware of the potential hazards associated with smoking. Countries such as Brazil, Thailand, Singapore, Hong Kong, Chile, Australia, and Canada have implemented this strategy, and research has found that it is especially effective for individuals with lower incomes [21].

Tobacco use is linked to cancer and other non-cancerous severe illnesses. These illnesses can be life-threatening and life-limiting. Some examples of these illnesses include coronary heart disease, atherosclerosis, peripheral vascular disease, chronic bronchitis, and emphysema [28].

Cigarette packs in India now feature health warnings with pictures of diseased lungs or radiographs of lungs with cancer. This is due to the prevalence of "beedi" use in India, which is a smoked product made of processed tobacco flakes hand-rolled in a tendu leaf. Because of the significant health risks associated with beedis, it is crucial to communicate the harmful effects of these products effectively [21,22].

Tobacco companies have long used packaging to promote brand loyalty and users' perceptions of themselves, especially among young people. It is important to keep in mind that adolescents today are likely to be the regular customers of tomorrow. Studies have shown that the majority of smokers begin smoking while they are still in their teenage years. As such, it is essential to put measures in place to reduce the impact that marketing has on teenagers. One way to do this is by including warnings on cigarette packs that make it clear to young people the negative health impacts that smoking can have on them [1,29]. When a smoker opens a cigarette pack, they are presented with graphic and informative images, which serve to remind them of the potential health risks associated with smoking. This form of communication is cost-effective and can help to spread health awareness on a wide scale. The images are a powerful reminder for smokers to consider the consequences of their habits and to consider quitting. Additionally, non-smokers are exposed to these warnings as they are often left in plain view when not in use. This creates a constant reminder of the potential dangers of smoking [27].

The health warnings on cigarette packages in India have been changing regularly. Although they are more intense than previous warnings, they have yet to be proven effective [27]. Despite tobacco control policies, the number of juvenile and young tobacco users is rising, as are diseases, disabilities, and deaths associated with tobacco use [8,30]. Previous health warnings on cigarette packets were effective in raising awareness among smokers, but the size, area covered, and position of the pictures must now be studied to provide proof of a decrease in the quit rate [21]. Despite the fact that these health warnings on tobacco packaging are likely to reach all smokers in India, there needs to be more research into how successful these messages are at engaging the intended audience. It is essential to understand how well these warnings are understood and acted upon in order to ensure their effectiveness [27].

Graphic health warnings significantly impact cigarette smokers' intention to quit in India, with a rising trend [31]. Gupta et al. investigated the impact of briefly viewing a text warning versus a graphic warning and discovered that graphic health warnings were more efficacious than text-only warnings in lowering smoking intentions [21].

To gauge reactions to graphic health warning labels, two questions are typically asked: "How do you feel when you see the graphic warning?" and "How do you feel when you read the text warning?" Answers to these questions are typically given using words such as "worried," "not to start," "like limiting," "like giving up smoking," and "nothing" [5,18,27]. The study found that people who had seen the graphic health warnings were more likely to have a positive attitude toward not smoking. However, acquiring this knowledge will keep some people's smoking habits the same [27].

Awareness Regarding Graphic Health Warning

Research indicates that the majority of tobacco users (over 90%) have observed graphic health warnings, and they have reported that these warnings helped to educate people about the cancer-causing qualities of tobacco products [5,18,21,27,32]. People were aware of the adverse effects of tobacco use on health and had a favorable attitude toward graphic warnings. However, despite the warnings, they lacked the motivation to give up the habit, possibly because the graphic warnings lacked the power to influence them to quit using tobacco [27].

Understanding Regarding Graphic Health Warning

The study revealed that the majority of the participants noticed the warnings printed on the tobacco packets. They were able to comprehend what the warnings were communicating and believed that they potentially had a positive influence on smoking habits. In particular, pictures on the warnings were found to be more effective than just text in understanding the risks of smoking, with cigarette smokers better grasping the message than beedi smokers [17,21,28].

The design and size of graphic health warnings can have a strong influence on how effective they are. Warnings with visuals, such as pictures or graphics, are more likely to make an impact than those that only contain text. Such warnings are visible on the front of cigarette packages and can provide important health information to smokers and non-smokers alike. They can also raise awareness of the risks associated with smoking and encourage people to quit.

Ignorance of Tobacco Users Regarding These Graphic Health Warnings

Despite being aware of the adverse health effects of tobacco, respondents in a survey were still asked how they could continue to use tobacco and potentially develop head and neck cancer. Results of the survey indicated that less than half of the participants had seen any warnings about the risks [18]. Most of the respondents reported that they did not think they were using enough tobacco to cause cancer. Some said they had started using tobacco before the graphic health warnings were put on the products. They also admitted that they had not taken the warnings seriously or paid attention to them [5,27,28].

Contribution of Graphic Health Warning in Motivation to Quit

Several studies have tried to gauge the effects of health warnings on the commencement of smoking among young people, and only a minimal number of participants believed that the warnings would lead to quitting or cutting down on tobacco use amongst existing users [5,18,27]. It was clear that smokers were cognizant of the warnings, and none of them were swayed by them, though the warnings did motivate some to consider quitting [33].

Never Attempted to Quit

It is concerning to note that a considerable number of tobacco users are yet to attempt to quit the habit within the last year, despite the presence of graphic warning labels, which calls into question the effectiveness of these warnings. Additionally, a few non-tobacco users should have offered advice to quit after viewing the graphic warnings, showing a lack of social motivation required for a tobacco addict to quit on their part [27]. Around 50% of the people in the research sample were conscious of the health warnings and thought that they successfully discouraged those who had not used tobacco from the beginning [18].

Need for a Bigger and Clearer Image

The importance of the dimensions and placement of health warnings cannot be overstated when it comes to their effectiveness [6,34]. If warnings are made prominent and visible enough, smokers will be more likely to remember them and recognize the degree of risk associated with them. Placing the warnings prominently at the top of the principal display area ensures that they will be seen when cigarettes are displayed for sale, taken out of pockets or purses, and disposed of [6]. Table 1 depicts a summary of the perceived influence of graphical warnings on people [5,18,21,27,32].

**Table 1:** A Summary of the perceived influence of graphical warnings on people

Study	Awareness regarding graphic health warning	Understanding regarding graphic health warning	Contribution of graphic health warning in motivation to quit	Ignorance of tobacco users regarding these graphic health warnings	Graphic health warning encourages to quit	Never made an attempt to quit	Need for bigger and clearer images
Vanishree et al., 2017 [5]	92.6%	39.85%	22.9%	39.7%	70%	-	40.3%
Kumar and Puranik, 2017 [27]	89.5%	81.5%	90.7%	66.7%	42%	37.1%	55.5%
Dahiya, et al., 2017 [32]	33.33%	64.2%	56.9%	57.3%	42.7%	48.3%	43.1%
Mullapudi et al., 2019 [18]	46%	-	32%	-	4%	-	-
Gupta et al., 2022 [21]	92.5%	54%	7.5%	-	9.5%	41%	-

Vigilance for compliance with statutory legislation for tobacco control

Despite the laws in place, tobacco products are still consumed for two primary reasons. First, many products, such as beedis, are made without following the guidelines set by public health departments. Cigarette users, on the other hand, are usually more aware of the regulations due to their production in licensed factories, which are subject to legal requirements. In comparison, beedis are often made in smaller-scale, home-based industries, meaning they can skirt around the legal framework [28,35].

It is essential to be vigilant in order to ensure that the laws in place are adequately enforced. Violators of government regulations that restrict the sale of tobacco products to individuals under the age of 18 should be subject to stern consequences. This is important to bear in mind since habits developed during youth tend to persist over a lifetime [33]. Anti-tobacco efforts must become mandatory in educational institutions, such as schools, colleges, and universities. Audio-visual aids should be employed to provide individual counseling, featuring examples of people who have been harmed by tobacco use. Health warnings should also be included in mass media campaigns, and the contact information of tobacco cessation helplines should be printed on tobacco product packaging [15,36].

The Government of India, through MoHFW, has updated the Cigarettes and Other Tobacco Products (Packaging and Labelling) Rules, 2008, with the Cigarettes and Other Tobacco Products (Packaging and Labelling) Third Amendment Rules, 2022. These new rules will take effect on December 1, 2022, and will include a set of specified health warnings that will be valid for 12 months following the commencement date [37].

**Figure 1:**
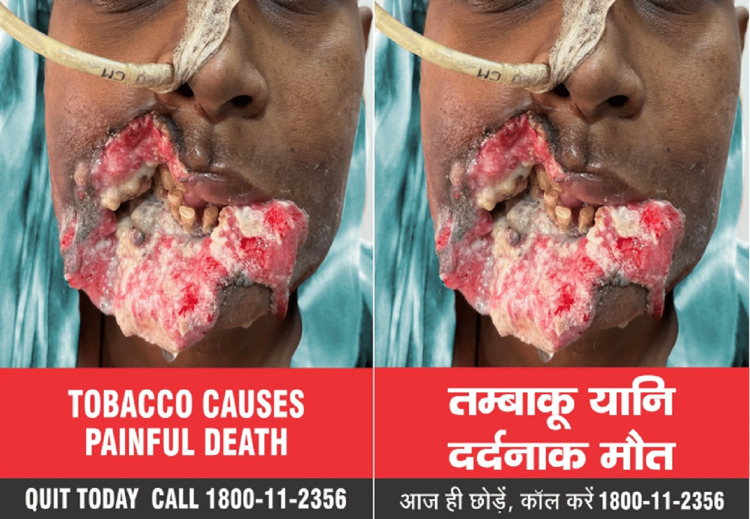
New specified health warning on tobacco products pack from December 1, 2022. This came into effect from December 1, 2022, and will remain valid for 12 months [37]. Data source: Available in the public domain [37].

**Figure 2:**
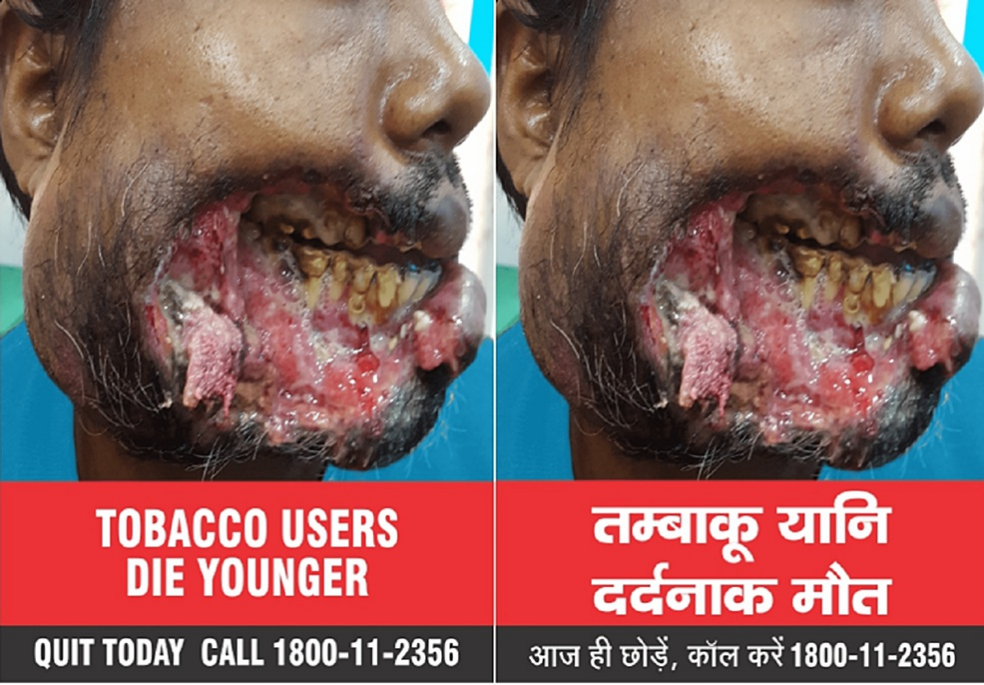
New specified health warning on tobacco products packs with effect from December 1, 2023. This health warning will become active with effect from December 1, 2023 [37]. Data source: Available in the public domain [37].

Starting December 1, 2022, all tobacco products have the image of a man with the words "TOBACCO CAUSES PAINFUL DEATH" printed on the packaging as shown in (Figure 1). By December 1, 2023, a new image (Figure 2) with the words "TOBACCO USERS DIE YOUNGER" must replace this. Whoever makes, supplies, imports, or distributes tobacco products must make sure that the package includes the appropriate health warnings. Not following this rule is a punishable offense under Section 20 of the Cigarettes and Other Tobacco Products (Prohibition of Advertisement and Regulation of Trade and Commerce, Production, Supply, and Distribution) Act, 2003, and can result in imprisonment or a fine [37].

This article's limitation is that it only uses studies from databases such as PubMed and Google Scholar, even though there may be many additional relevant studies in other databases that we are not aware of.

Recommendation

There should be more straightforward and more eye-catching labels to make graphic warnings on tobacco products more effective. Consideration should be given to including local and regional languages in India, in addition to text messages in English, as this is the language understood by most people. It is also beneficial to have warnings on each individual cigarette piece. In India, considering a large portion of the population is illiterate or not fluent in English, the graphic warnings on tobacco products must be self-explanatory, with no attractive qualities. Additionally, the advertisement boards at tobacco retailers should have a clear, large, graphic warning sign.

Schools should provide educational resources to students and parents about the health risks of tobacco use, implement and enforce school-wide tobacco bans, prohibit tobacco advertising and marketing in school, educate students about the harms of tobacco use, utilize tobacco health warning labels, increase access to cessation services, utilize media campaigns, create peer-to-peer programs, provide support for teachers, and partner with local health organizations to provide resources and support to the school community on tobacco prevention.

One way tobacco non-users can provide social support to someone trying to quit smoking is to offer emotional support and encouragement. Showing your friend that you care and are there for them no matter what can have a powerful effect. Listen to their challenges and help them find positive ways to cope with cravings. Offering emotional support, suggesting activities such as hobbies, providing incentives and resources, monitoring their progress, and celebrating milestones can help someone quit tobacco. Doing these things can make a huge difference in someone's life and help them lead a healthy life.

More qualitative studies, such as focus group discussions and in-depth interviews with all stakeholders to uncover the motivating and demotivating factors associated with tobacco use, should be conducted. The insights gained from these qualitative studies can then be used to strengthen further existing guidelines and legislation related to tobacco control.

## Conclusions

The results of this study show that only one in five non-tobacco users were deterred from starting to use tobacco after seeing the graphic health warnings on tobacco packs. Research has shown that tobacco users have an understanding and generally favorable opinion of the graphic warnings on cigarette packages. However, they need more motivation to discontinue their habit, even with the warnings in place. This suggests that the quality of the warnings should be improved in order to make them more effective in discouraging people from using tobacco by making them more visible, with the use of more shocking images, text in the native language of the person viewing it, and additional information about the potential health risks associated with it. The tobacco crisis is devastating and far from over but the gains achieved till date can be further prevented with suitable measures.
